# The objective measurement of physical activity and sedentary behaviour in 2–3 year olds and their parents: a cross-sectional feasibility study in the bi-ethnic Born in Bradford cohort

**DOI:** 10.1186/s12889-015-2481-z

**Published:** 2015-11-11

**Authors:** Silvia Costa, Sally E. Barber, Noël Cameron, Stacy A. Clemes

**Affiliations:** Institute of Child Health, University College London, 30 Guilford Street, London, WC1N 1EH UK; School of Sport, Exercise and Health Sciences, Loughborough University, Ashby Road, Loughborough, Leicestershire LE11 3TU UK; Born in Bradford Cohort Study, Bradford Institute for Health Research, Bradford Royal Infirmary, Duckworth Lane, Bradford, BD9 6RJ UK; NIHR Leicester-Loughborough Diet, Lifestyle and Physical Activity Biomedical Research Unit, Loughborough University, Ashby Road, Loughborough, LE11 3TU UK

**Keywords:** Accelerometer, Objective measurement, Physical activity, Sedentary behaviour, Toddlers, Family, South Asian

## Abstract

**Background:**

The reported lower physical activity (PA) levels of British South Asians (SA) are suggested as a key influence in their increased risk of non-communicable diseases compared to their White British peers. Differences in objectively measured PA and sedentary behaviour (SB) between these ethnic groups have been observed during childhood (ages: 8–10 years). However, no information exists on objectively measured PA/SB in younger children, or how early in life differences in these behaviours emerge. Assessing PA/SB in the Born in Bradford (BIB) cohort study provides an opportunity to address such gaps in the literature, but previous studies have found recruiting and retaining SA participants challenging, and the feasibility of using accelerometers with SA children and parents is unknown.

This study investigated the feasibility of recruiting and objectively measuring the habitual PA/SB of 2–3 year old SA and White British children and parents from the BIB study.

**Methods:**

Families were informed about the study during routine BIB assessments. Consenting families were visited at home for anthropometry measurements, interviews, material delivery and collection. Participants (child and parents) were instructed to wear the ActiGraph GT3X+ for 8 days. Descriptive statistics were computed, and ethnic differences tested (Chi-square) for recruitment uptake and compliance.

**Results:**

160 families (30 % SA) provided contact details, and 97 (22 % SA) agreed to enter the study. White British families showed lower refusal and higher intake into the study than SA (*p* = 0.006). Of 89 children issued with an accelerometer, 34 % complied with the 8-day protocol (significantly less SA; *p* = 0.015) and 75 % provided enough days (≥3) to assess habitual PA/SB (no ethnic differences). Parental rates of compliance with the protocol did not differ between ethnicities. Issues experienced with the protocol and accelerometer use, and successful implementation strategies/procedures are presented.

**Conclusions:**

Although greater efforts may be required to recruit SA, those consenting to participate were as likely as White British to provide enough data to assess habitual PA/SB. The issues and successful strategies reported in this feasibility study represent valuable information for planning future studies, and enhance recruitment and compliance with accelerometer protocols in SA and White British toddlers and parents.

**Electronic supplementary material:**

The online version of this article (doi:10.1186/s12889-015-2481-z) contains supplementary material, which is available to authorized users.

## Background

South Asians are at a higher risk of developing non-communicable diseases such as coronary heart disease, insulin resistance and diabetes [1]. This increased risk has been reported in both adults [2, 3] and children [4, 5] who emigrated from South Asian countries, and seems to persist in subsequent generations already born in the country where they migrated to [6]. Authors have reported that ethnic differences in adiposity [6], socioeconomic status (SES), insulin resistance parameters or metabolic syndrome [3] do not account for the excess risk for non-communicable diseases in South Asians compared to White British people. It has been suggested that the lower physical activity (PA) levels of South Asians play a major role in their worse metabolic profile and higher risk for non-communicable diseases [7–10]. Owen et al. [9] have reported that 9–10 year old South Asian children registered 905 fewer steps (95 % confidence interval (CI): 624–1187), spent 39 min more (95 % CI: 26–52) in sedentary behaviour (SB), and five minutes less (95 % CI: 2–7) in moderate-to-vigorous PA (MVPA) than their White British peers (all *p* < 0.001), as measured by the ActiGraph GT1M accelerometer. Using a combined heart rate and movement sensor, a more recent study by Eyre et al. [11] has also reported that South Asian children (8–9 years) were less likely to meet PA guidelines and less active than White British children during both weekdays and weekends.

It has been urged that the prevention and management of the metabolic syndrome, coronary heart disease and diabetes in South Asians should begin early in childhood [8, 12], with a particular focus on lifestyle changes including increases in PA [5]. However, there is no information on objectively measured PA and SB levels of South Asian children younger than 8 years of age. Consequently, it is unknown how early in life these differences in PA and SB begin to appear between South Asian and White British children. It is also not known which factors influence young South Asian children’s PA/SB levels, or the extent to which differences in activity levels influence the emergence of ethnic inequalities in the precursors of non-communicable diseases (e.g. increased adiposity and insulin resistance) [4, 9].

The Born in Bradford (BIB) cohort study is a longitudinal birth cohort study involving a mostly bi-ethnic sample of over 13,000 families of children born at the Bradford Royal Infirmary between 2007 and 2010, which aims to identify the factors that contribute to health and those that influence health disparities in people from South Asian and White European origins [13, 14]. Given the ethnic composition of the sample, the BIB cohort study presents a remarkable opportunity to address this gap in the literature, and to investigate the potential influences that parental PA and SB levels may have on their offspring’s activity behaviours throughout childhood [15]. Collecting objectively measured data for enough days from both South Asian and White British toddlers and their families is crucial to assess any ethnic differences in PA/SB levels, but this can be challenging. Particular difficulties in recruiting South Asians into research have been previously highlighted in the literature [16, 17], and South Asian women have reported finding certain activity monitors as intrusive [18, 19]. Despite the increasing emphasis on the need to include ethnic minorities in research, information about the best practice and rates of recruitment in British South Asian populations is scarce [17], and no studies besides that of Pollard & Guell’s [18] have reported on the feasibility and acceptability of South Asians (adults or children) wearing accelerometers in PA measurement studies. Furthermore, families taking part in the BIB cohort study are already required to contribute to a large number of assessments [13]; it is possible that the additional burden resulting from the introduction of PA and SB measurement may not be acceptable to participating families, but this has not yet been assessed.

In a previous study, the ActiGraph GT3X+ was found to be the most accepted accelerometer for use in South Asian and White British children and parents, as well as the accelerometer for which mothers identified the least amount of potential issues for use with their toddlers [19]. However, this was assessed only through mothers’ opinions, and not through the actual experience of wearing the ActiGraph GT3X+. Furthermore, children’s acceptability cannot be the only deciding factor when choosing the adequate activity monitor for a study. Arguably, the most important factor regarding monitor selection is the required outcome measure, along with device reliability and validity [20]. The ActiGraph GT3X+ has been shown to be a feasible and valid tool, calibrated to measure PA and SB in 2–3 year olds [21]. Testing the feasibility of using a previous ActiGraph model (GT1M) with Belgian 1–2 year olds, van Cauwenberghe et al. [22] reported that 64 % of children provided enough valid days (≥3) to be included for further analyses. Additionally, 83 % of parents reported their toddler found it “not unpleasant and not pleasant” to wear the accelerometer, with none finding it “very unpleasant” or “umpleasant” [22]. However, the feasibility of wearing the ActiGraph GT3X+ for enough days to accurately assess the habitual PA and SB of 2–3 year olds of different ethnicities has not yet been tested. Therefore, the aims of this study were to assess the feasibility of recruiting and measuring the habitual PA and SB of 2–3 year old South Asian and White British children and both parents participating in the BIB cohort study.

## Methods

### Sample recruitment

Participants were recruited by BIB staff during routine home visits or baby clinics, conducted with families taking part in the BIB1000 focus sample from the BIB study, which aims to investigate growth trajectories and identify modifiable risk factors for childhood obesity [14]. BIB1000 includes over 1700 families who agreed to have additional measurements taken at six, 12 and 18 months, 2, 3 and 4 years [14]. The BIB1000 focus sample is composed by a sub-set of participant families who have consented to having additional data collected regarding growth, diet, feeding practices, PA, screen viewing and sleep [14]. Bi-lingual BIB staff (of both White British and South Asian ethnicities) showed the participant information sheet while briefly explaining the study to the parents in their preferred language, and filled in a contact sheet for those who showed an interest in taking part. The participant information sheet briefly described the study procedures, what was required from participants, the importance of understanding PA and SB levels and the factors influencing these, and contained contact details for the main investigators. Participant information sheets were available both in English and Urdu, to facilitate the recruitment of non-English speaking South Asian families. The contact sheets were collected from the BIB offices on a regular basis by the lead researcher for this physical activity measurement study, contact details were extracted into a database, and each family was assigned with a unique study ID. Parents were contacted by telephone at their preferred days and times, unless email was the preferred method of contact stated (in which case the first contact was via email). During the telephone call, the study procedures were explained with greater detail, and parents were provided with the opportunity to ask questions. For those who agreed to participate, current address was confirmed and a date and time was set for the first home visit. All recruitment telephone calls were made following a template guide, to ensure that no important information was missed. If there was more than 1 week between the telephone call and the first home visit, participants were also asked if they would like a reminder text message or telephone call to confirm if the scheduled date and time were still suitable. For participants who declared that they would like to think about the decision to take part in the study, the researcher asked for permission to call them again and the best time to do this.

### Data collection procedures

After the recruitment telephone call, each family who agreed to take part in the study was assigned with a “family pack” consisting of: one checklist; one first home visit guide; parent and child informed consent forms; one anthropometric measurement sheet; one activity diary; one mother’s questionnaire form; one “end of data collection” interview; one magnet reminder to wear the accelerometers; one extra belt for the child’s accelerometer; and one accelerometer per participant family member. Each accelerometer was identified with a label which allowed participants to clearly identify each family member’s unique accelerometer, thus reducing the possibility of unintentional switches between children and their parents. The additional belt was included to allow parents to quickly replace the belt in case the original one needed washing (e.g. from issues resulting from *potty training*, as experienced by one family). The additional belt also enabled children to wear the ActiGraph GT3X+ during aquatic activities, providing a dry belt to place the accelerometer back on straight after the activity, thus avoiding loss of data because of waiting for the original belt to dry.

During the first visit, parents were reminded of study procedures, given the opportunity to ask questions, and requested to sign informed consent forms for themselves and their children. After formal consent was given, parents’ and children’s anthropometric measurements were taken by the first author, including height, weight, and waist circumference, hip circumference (parents only), and subscapular and triceps skinfolds (children only). Height was measured to the nearest one mm using a portable stadiometer (Holtain Ltd, Crosswell, UK), with a wooden standing platform placed on a firm horizontal surface. Weight was measured to the nearest 0.1 kg on a Tanita weighing scale (Tanita, model BC 418 MA, Tokyo, Japan) placed on a firm horizontal surface, with participants being barefoot and free of heavy clothing (e.g. outdoor jackets, sweat-shirts or work trousers). Waist circumference was measured with a Seca measuring tape to the nearest one mm at the level of the umbilicus directly over the skin, when this was allowed by the participants. In case participants considered this to be embarrassing or unsuitable (particularly due to cultural sensitivity in South Asian families), they were asked to either change into lighter clothing or remain with the thinnest possible layer of clothing for the measurement to be taken, with care to remove any bold creases that would introduce further error into the measurement. Whenever it was not possible to perform the measurements on the first home visit, these were taken during the last home visit or on a third appointment specifically arranged for this purpose up to 8 days after the last day of data collection. At the end of this first visit, the time and date for the last home visit was scheduled and participants were asked if they would also like to receive a text message during the first three days of data collection to remind them to wear the accelerometer. If participants requested this, mobile phone contact and usual wake-up times for both weekdays and weekend days were recorded, and a standardised text message reminder was sent roughly five minutes after reported wake-up times. All participants were advised to contact the author via email or telephone if there were any concerns or issues regarding any study materials or protocols during data collection.

During the second home visit, all monitors and study documents were collected, and any missing anthropometric measurements were taken. After completion of the questionnaire, a semi-structured “end of data collection” interview was conducted with the mother to assess if there were difficulties in wearing the accelerometer for both parents and the child, filling in the activity diary or the questionnaire, whether the week of data collection had been a “typical week” or more/less active (and why), and also if the child had been ill during that week. This was an informal one-to-one interview with several open-ended questions to allow parents to provide as much detail as possible on any issues and successful strategies used (see Additional file 1 for copy of interview guide). Upon completion of the study, all families were sent written reports informing them of the average PA and SB levels of the children and parents. These reports also contained current PA and SB guidelines [23] and tips on how families could achieve these recommendations.

Participants’ SES was assessed using the 2010 English Index of Multiple Deprivation (IMD) [24] with participants’ home postcodes. There are 32,482 postcode areas in England; the English 2010 IMD combines a range of economic, social, and housing indicators into a single deprivation score for each postcode area, thus allowing the ranking of areas from 1 to 32,482, according to their level of deprivation [24]. To facilitate interpretation, the ranking was converted to percentages where 0 % represents the most deprived area and 100 % represents the least deprived area.

### Physical activity and sedentary behaviour measurement

**Fig. 1:**
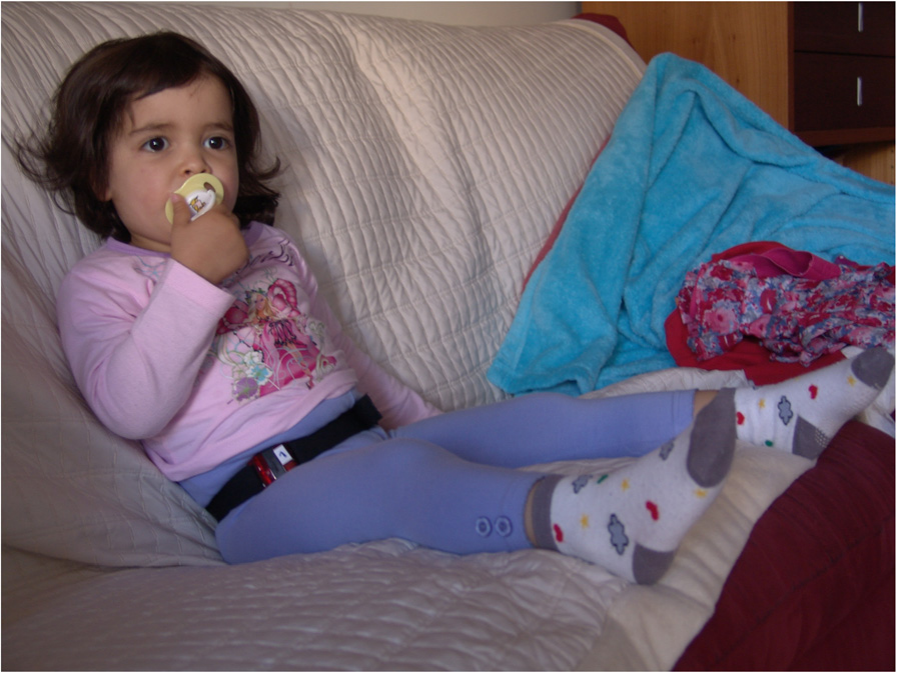
Accelerometer placement on a 3-year old child

Children’s and parents’ PA and SB were measured with the ActiGraph GT3X+ (The ActiGraph, Pensacola, FL, USA), during waking hours over eight consecutive days. The ActiGraph GT3X+ was set to record data at 60Hz, which was the maximum frequency possible for nine consecutive days of data collection. This nine-day period was programmed to give families the opportunity to still comply with the eight-day protocol if they forgot to wear the monitors on the first day. Each device was programmed to start recording at 5:00 am on the first day (the day after first home visit), and stop recording at 5:00 am on the 10^th^ day (i.e. nine full 24-h days). At the first home visit, parents (most commonly mothers) were instructed and shown how to place the ActiGraph GT3X+ on themselves and the children at the level of the anterior superior iliac spine, underneath or on top of clothes, and to place it consistently on the same side of the body (see Fig. 1 depicting the accelerometer on a 3-year old child). We did not give strict guidance for placement on the left or right side of the body, to enable participants to choose the most comfortable side for them, and avoid switching sides during data collection (e.g. if participants mistakenly started wearing the device on the “wrong side” and corrected the placement half way through the collection period). Parents were advised to place the devices underneath children’s clothes, to avoid any issues resulting from children’s curiosity (e.g. removal of the device, or erroneous acceleration data resulting from the child playing with the device). Parents were instructed to place the accelerometers on themselves and their children after waking up in the morning, remove them at the time of going to bed, and to note down in the activity diary any other periods when the accelerometer was removed with the corresponding reason. Additionally, they were asked to record in the activity diary any day-time sleep (i.e. naps) that children may have during the data collection period.

After collecting the monitors at the second home visit, the raw accelerometry data (*.gt3x file) was downloaded and analysed in the proprietary software ActiLife (v.6). The raw accelerometry files were then transformed into an *.AGD file, displaying the acceleration counts by 5- and 60-s epochs (for children and parents respectively), using the low frequency extension filter. Due to the scarce research defining the correct criteria specifically for toddlers [25], wear-time validation and day inclusion criteria was undertaken in agreement with procedures used previously in preschoolers [26], toddlers [22], and infants [27]. Non-wear time was determined as ≥10 consecutive minutes of zero counts, which has been considered adequate for the typical activity patterns of this age group [28]. Following wear time validation, children’s *.AGD files were scanned to assess what time the accelerometer was placed on and taken off, thus defining the start and end of each day. The start of a day was set at the first consecutive minute of acceleration data higher than zero, and the end of a day was defined as the last epoch with counts higher than zero before a consecutive ≥180 min of zero counts. A valid day was defined according to the 70/80 rule [22, 27, 29]: 80 % of the period during which at least 70 % of the study population has recorded accelerometer data. Only days with ≥3 h and ≤18 h of valid wear time were included in this calculation to ensure that implausible wear times would not influence the definition of a valid day or the subsequent assessment of SB and PA levels [25, 30]. Since at least 70 % of the sample had ≥587.82 min of valid acceleration data, a valid day was defined as one containing ≥470.26 min of valid acceleration data (similar to previous studies with children aged ≤3 years) [22, 27]. The number of days needed for a reliable estimate of habitual SB and PA in children younger than 3 years has not yet been agreed on [24]. After investigation published elsewhere [31] regarding the minimum number of days needed to get reliable measurements of habitual PA/SB, which followed procedures similar to those used by Hinkley et al. [30] in preschoolers: children with ≥3 days of valid data were considered to provide enough data to assess habitual time spent in SB and PA. These three days did not necessarily include both week and weekend days, due to previous investigation showing no difference in PA/SB between week and weekend days in this sample [31].

For parents, accelerometry data cleaning procedures followed those previously employed in the literature [32, 33]. Non-wear time was defined as ≥60 min of consecutive zero counts, and a day was considered valid if wear-time was ≥600 min. Parents with ≥3 valid days were considered to provide enough data for inclusion in studies assessing habitual PA/SB. All procedures were reviewed and approved by the University’s Ethical Advisory Committee prior to the start of the study.

### Statistical analysis

All scale variables were checked for normality of distribution by visual inspection of histograms and formally tested. Analyses were run in Stata (v.12) and SPSS (v.19) statistical software packages. Descriptive statistics (means, medians, standard deviation (SD), inter-quartile ranges (IQR), and percentages) were computed to describe the sample of provided contacts, recruitment uptake, compliance with the study protocol of eight days of wearing the accelerometer, and the sample providing ≥3 valid days of accelerometry data. Differences in these four variables between ethnicities, mother and father, and child’s sex were assessed with the Chi-square (χ^2^) or Fisher’s Exact test (when >25 % of cells had <5 expected counts). Mann–Whitney U or unpaired t-tests (when data were normally distributed) were used to compare the SES between those who accepted or refused to take part; those complying versus non-complying with 8-day protocol; and those providing ≥3 days of valid accelerometry data or not.

## Results

### Recruitment uptake

A total of 160 families provided contact details during recruitment at BIB clinics or home visits. When contacted by the lead researcher to discuss the study further, 70.3 % agreed to participate in the study, and only 17.4 % refused. A further 12.3 % requested to be contacted again at a later date, and/or did not answer the contact attempts made by the author. Roughly one third of families who provided contact details were South Asians, and half of the sample belonged to the 20 % most deprived SES. Further details can be seen below in Table 1.

**Table 1:** Recruitment uptake (in total and by ethnicity, n [%])

	Total	White British	South Asian
Families	160 (100.0 %)	112 (70.0 %)	48 (30.0 %)
IMD rank^a^	18.30 % (6.1–41.0 %)	24.55 %* (6.1–44.9 %)	8.80 %* (6.3–22.6 %)
Recruitment			
Accepted	97 (70.3 %)	76* (77.6 %)	21* (53.5 %)
Refused	24 (17.4 %)	11* (11.2 %)	13* (32.5 %)
Contact again/no return to contact	17 (12.3 %)	11* (11.2 %)	6* (15.0 %)

*IMD* Index of Multiple Depravation

*significant difference between ethnicities (*p* ≤ 0.006)

^a^Median (inter-quartile range)

The IMD differed significantly between ethnicities (higher in White British; *p* < 0.001), but not between those who accepted or refused to take part in the study (*p* = 0.461). During the telephone contacts, there was a significantly lower refusal rate and higher proportion of uptake into the study by White British in comparison with South Asian families (χ^2^ = 10.26, degrees of freedom [df] = 2, *p* = 0.006).

### Compliance of children

In total 89 children (53.9 % boys; 25.8 % South Asians) were set to start data collection. Due to various reasons (e.g. incompatible schedules, consecutive re-scheduling, and parents in process of divorcing), it was not possible to undertake data collection for eight children whose parents accepted to take part during the study timeline. Only 33.7 % of those who were set to start data collection actually complied to the instructed eight consecutive days of data collection, with a significantly higher proportion of White British complying with the protocol than South Asians (χ^2^ = 5.93, df = 1, *p* = 0.015). There were no significant differences between sexes or South Asian and White British in the number of children providing ≥3 valid days (p ≥ 0.2). There was, however, a significant difference between ethnicities in daily wear time. South Asian children wore the accelerometer for a median of 696 min per day, whereas White British children wore the accelerometer for a median of 642 min per day (*p* = 0.017). Full details about wearing and compliance rates can be seen in Table 2.

**Table 2:** Compliance of children with data collection protocol (by sex and ethnicity)

	Total	Boys	Girls	White British	South Asian
Participants	89 (100.0 %)	48 (53.9 %)	41 (46.1 %)	66 (74.2 %)	23 (25.8 %)
IMD rank^a^	17.34 % (5.9–41.5 %)	18.8 % (8.2–41.6 %)	12.6 % (5.4–40.8 %)	21.6 %* (5.9–44.4 %)	8.2 %* (5.7–12.7 %)
Days worn^a^	7 (5.3–8)	7 (6–8)	7 (5–8)	7 (5.8–8)	6 (4.5–8)
Compliance with 8-day protocol					
Complied	30 (33.7 %)	16 (33.3 %)	14 (34.1 %)	27* (40.9 %)	3* (13.0 %)
Enough valid data (≥3 days)					
Yes	67 (75.3 %)	36 (75.0 %)	31 (75.6 %)	52 (78.8 %)	15 (65.2 %)

*IMD* Index of Multiple Depravation

*Significant difference between ethnicities (*p* ≤ 0.017)

^a^Median (inter-quartile range)

The IMD ranks of both South Asian and White British families who provided contact details (Table 1) were similar to the IMD ranks of the 89 children who underwent data collection (Table 2).

### Compliance of parents

In total, 133 parents (60.1 % mothers; 23.3 % South Asians) had a first home visit and were set to start data collection. There were no significant differences between mothers and fathers or between ethnicities in the number of individuals providing ≥3 valid days. Significantly more mothers complied with the 8-day protocol than fathers (χ^2^ = 6.99, df = 1, *p* = 0.008). Full details can be seen in Table 3.

**Table 3:** Compliance of parents with data collection protocol (by parental figure and ethnicity)

	Total	Mother	Father	White British	South Asian
Participants	133 (100.0 %)	80 (60.1 %)	53 (39.9 %)	102 (76.7 %)	31 (23.3 %)
IMD rank^a^	16.6 % (5.7–41.7 %)	15.8 % (5.6–41.5 %)	18.8 % (5.8–43.7 %)	23.4 %* (5.5–44.4 %)	10.1 %* (5.7–118.0 %)
Days worn^a^	7 (6–8)	8^**^ (6–8)	7^**^ (5–8)	7 (5–8)	7 (6–8)
Compliance with 8-day protocol					
Complied	54 (43.9 %)	40^**^ (54.1 %)	15^**^ (30.0 %)	44 (45.4 %)	11 (40.7 %)
Enough data (≥3 days)					
Yes	112 (91.1 %)	70 (94.6 %)	43 (86.0 %)	87 (89.7 %)	26 (96.3 %)

IMD - Index of Multiple Depravation

*Significant difference between ethnicities (*p* = 0.005); ^**^significant difference between mother and father (*p* ≤ 0.018)

^a^Median (inter-quartile range)

There were 59 families (22.0 % South Asian) where both children and ≥1 parent provided ≥3 valid days of accelerometer data, with no difference between ethnicities (*p* = 0.2).

## Discussion

The current study assessed the feasibility of introducing the additional measurement of toddlers and parents’ habitual PA and SB into the BIB cohort study, exploring the possible differences in the recruitment and compliance rates between South Asian and White British families. Although there was a significantly higher rate of White British families who provided contact details (70 % versus 30 %) and agreed to take part in the study (77.6 % versus 53.5 %), there was no difference in the number of South Asian and White British parents and children who provided enough data for the assessment of habitual PA and SB (see Tables 2 and 3). This suggests that the difficulty may be indeed in the recruitment of South Asian participants, as previously reported in the literature [16, 17], but not due to a lack of compliance or providing insufficient data once they have agreed to take part in the study.

Rooney et al. [17] identified several barriers to the recruitment of South Asians into research, including unfamiliarity with the research process, fear of the unknown, competing priorities (e.g. employment or childcare), potential language or communication issues, and the lack of a direct and personalized approach from researchers (considered by Rooney and colleagues as the main barrier). Although the current study attempted to recruit participants through BIB staff, with whom the families were already familiar and have an ongoing trust relationship, recruitment of South Asian families particularly could have benefited from the establishment of further partnerships with religious and local South Asian organisations, which was a successful strategy used previously by Douglas et al. [16].

Although three valid days has proved to be enough for the reliable assessment of habitual PA and SB (ICC ≥0.80) in 2–3 year old South Asian and White British children [30], data collection periods may need to last longer than this. For 10 out of the 37 children who did not comply with the 8-day protocol but still provided ≥3 valid days, it took between four and five days to reach this minimum. In one case, it took seven days for the child to add up three valid days. It may be sensible to set the data collection period at a minimum of five days in future studies assessing the habitual PA and SB of 2–3 year olds, to allow for the exclusion of two invalid (or non-wear) days and still obtain the minimum three days needed for inclusion in the study.

### Issues, reasons for non-wear and suggestions reported by parents

Nearly all parents reported that wearing the accelerometer was not difficult for their toddlers. For some of the children who provided ≥3 valid days, parents reported that wearing the ActiGraph GT3X+ was “*fun*” and that on many days it was the child who reminded the parent to place the accelerometer on in the morning. This is in line with the study by van Cauwenberghe et al. [22], where most parents reported that wearing the accelerometer was “not unpleasant and not pleasant” for their toddlers. The main issue reported by parents was the unwillingness of some children to wear the accelerometer, even after a few days and several strategies used by the parents to try to convince them. The latter was the reason given for the majority of children who started data collection but did not register any valid days of wearing the accelerometer, in both South Asian and White British families. In most cases, parents had expressed uncertainty about the willingness of children to wear the monitor during recruitment or the first visit, reflecting their experiences with other accessories which their offspring refused to wear in daily life (e.g. belts or hair accessories). Therefore, the loss of these participants was not unexpected – it is advisable for researchers to provide those parents reporting doubts about child’s willingness to wear the activity monitor with several possible strategies to try to tackle these more challenging situations (e.g. wearing monitor under the clothes; competing with a parent/sibling to see who can wear it for longer). Two devices could not be retrieved from a family, even after the last home visit and two phone calls, due to the participants losing the accelerometers. There were also four children for whom the parents reported no issues with wearing the ActiGraph GT3X+ but who did not provide any valid days. Two children used the accelerometer as a “hide-and-seek” game, which resulted in no valid days registered because of temporary misplacement of the device, or too many daily occasions of non-wear time. Similar issues due to child curiosity had also been reported by van Cauwenberghe et al. [22]. Three further children did not wear the accelerometer due to personal issues of parents (e.g. divorces or extra unexpected work load), which made it difficult to remember to wear the accelerometer during the data collection period, or just too burdensome at the time. Such issues are likely to happen in other settings, and should be taken into account when calculating sample sizes needed for future studies.

In agreement with speculations previously made in focus groups [19], some mothers reported that their children had occasionally played with the ActiGraph GT3X+ when its presence was noted or refused to wear the device. As mentioned above, this resulted in the lack of valid days for some children. However, two children were recovered for inclusion after a second attempt or as a result of prolonging the data collection period. Mothers reported using strategies such as encouraging their child to compete with them to see who would wear it for longer, or using a reward scheme for wearing the accelerometer from morning until bed time. Similar strategies may be useful for future studies using activity monitors to measure the habitual PA and SB of such young children.

There were also two suggestions made by the parents regarding the use of the ActiGraph GT3X+. One was regarding the use of the accelerometer with the parents: some parents suggested that using a clip instead of a belt might be easier and more comfortable for individuals whose professions already require the use of specialised belts and accessories (e.g. policemen or construction workers). The other was regarding the use of the accelerometer with the children: it was suggested that placing the accelerometer underneath the clothes (as advised during the first visit) was the best strategy to avoid the children noticing its presence, and playing with the device due to their inherent curiosity. Importantly, the reported issues were common to both ethnic groups.

### Hardware issues

Additionally to the loss of data resulting from practical issues, there was partial data loss from two participants due to faulty memory of two devices. An older batch of ActiGraph GT3X+ showed a shortening in battery life towards the final data collection rounds (regardless of the complete recharging of batteries prior to every round), which in two cases resulted in the loss of the last day. This issue with battery life results from the repeated usage of the accelerometers. It is advisable to ensure the complete recharge of batteries before all data collection rounds, to avoid the loss of valuable data. If low temperatures are expected during the programmed data collection period, and the latter is longer than six days (minimum time recorded before device stand-by due to low battery), an alternative option could be to provide participants with a second accelerometer unit half-time between monitor delivery and collection..

### Other successful strategies

Providing participants with a report of their PA levels and PA guidelines at the end of data collection was valued by all parents – all 12 families who returned short feedback forms confirmed that the information provided in the report was clear and useful. Additionally, providing this report was one of the main reasons for some parents to take part in this feasibility study. The magnet reminder was reported by most families as a good strategy to remind parents to wear the ActiGraph GT3X+ soon after waking up. The text messages at time of waking up during the first three days was reported as very useful by all families who requested it, with over one third of mothers reporting it as crucial for complying with the protocol, without which they would not have remembered to place the monitor on themselves and their children on the first day. Programming the ActiGraph GT3X+ with one extra day allowed three families to still comply with the 8-day protocol, or at least enough days to be included in studies assessing the habitual levels of PA and SB in both the toddlers and parents. Similar issues with parents forgetting to place the accelerometer on their child have been reported before, leading to the exclusion of five out of 47 children due insufficient valid days collected in the study by van Cauwenberghe et al. [22]. Therefore, we advise researchers to employ at least some of these successful strategies (or alternative ones) in future studies, to minimise the risk of excluding children due to such issues.

Providing participants with a contact telephone number or email which they can use when doubts arise regarding the data collection protocol is also advisable. In the current study, several mothers contacted the author during the first days of data collection regarding different issues, such as making sure the device should not have lights on while collecting data (as they were programmed to flash while waiting to start data collection), or asking for suggestions of strategies to encourage the child to wear the accelerometer. The clarification of such doubts and reassurance provided by the first author was appreciated by the mothers, and reported by some as very useful for the success of using the accelerometer for enough days with the children. The use of a check-list with all the materials/documents needed before each visit was crucial to avoid the loss of important data due to forgetfulness. Collecting parents’ opinions and suggestions about study documents, materials and protocols is extremely important and advised, preferably with open-ended questions. The latter allows participants to fully describe any issues that they may have faced and any strategies used to cope with them. This information is valuable to enable the refinement of study procedures and documents, minimising participant burden, and promoting better compliance with future study protocols.

This study is not without limitations. First and foremost, the recruitment rates and intake into the study are inherently linked to the BIB study and the procedures used for recruitment and retention of participants. Because this was only a feasibility study and a “satellite research project” within the core BIB cohort study, special care was taken to avoid creating an extra burden for participants, which could lead to the drop-out of families from the main BIB study. Thus, similar studies that are not attached to a cohort study may be able to employ different or additional strategies to improve recruitment and retention rates (e.g. recruitment through additional community or religious organisations). Secondly, because BIB staff were only asked (not obliged) to publicise and provide details of this study, and requesting details about the families that had been approached was considered too burdensome by BIB staff – we were unable to collect details regarding the ethnic composition and total number of families approached to participate in this study. This precluded the calculation of rates of families (overall and by ethnicity) providing contacts and accepting/refusing to participate within the BIB1000 focus sample. Thirdly, many of the issues identified during data collection may be very specific to the sample of the current study and to the particular accelerometer used. The acceptability and ease of wearing the accelerometer, and compliance with protocol will not necessarily be the same if a different monitor (with different size, shape, and placement location) was used. However, the majority of the practical issues noted are transferable to the wider population and to varied settings, and the suggestions and strategies employed would be relevant in any study attempting to assess the habitual PA and SB of toddlers with activity monitors in general.

## Conclusions

To our knowledge, this was the first study to investigate the feasibility of objectively measuring PA and SB in South Asian and White British toddlers and their parents. From our results, it was feasible to recruit and measure the habitual PA and SB of both South Asian and White British toddlers and their parents. More White British than South Asian families were recruited and agreed to take part in the study, but both ethnicities showed similar rates of toddlers and parents providing enough data for the assessment of habitual PA and SB. This indicates that a greater effort may be needed during the recruitment stage to enrol adequate numbers of South Asian participants, but not in later stages of the studies in order to get enough valid data to assess habitual activity behaviours. Additional opportunities for the direct recruitment of South Asian participants, such as partnering with trusted institutions, may help to overcome some of the barriers previously reported in the literature [17], thus increasing the chances of recruitment and intake of South Asian families into a study. The issues and suggestions resulting from this feasibility study can be considered when planning future studies to enhance the recruitment and compliance to measurement protocols with young South Asian and White British children and their parents.

## Additional file

Additional file 1:
**PDF document with copy of guide for the “end of data collection interview”.** (PDF 137 kb)

## Abbreviations

CI: Confidence interval
IQR: Inter-quartile range
MVPA: Moderate-to-vigorous physical activity
PA: Physical activity
SB: Sedentary behaviour
SD: Standard deviation
SES: Socioeconomic status
