# Supplementary material for: The objective measurement of physical activity and sedentary behaviour in 2–3 year olds and their parents: a cross-sectional feasibility study in the bi-ethnic Born in Bradford cohort
Source: BMC Public Health. 2015 Nov 11;15:1109. doi: 10.1186/s12889-015-2481-z (PMC4641382; doi:10.1186/s12889-015-2481-z)
Supplement: Additional file 1: — PDF document with copy of guide for the “end of data collection interview”. (PDF 137 kb) [file 12889_2015_2481_MOESM1_ESM.pdf]

BORNINBRADFORD
For a Healthy Future

Loughborough University

Physical Activity and Sedentary Behaviour in a Bi-Ethnic Population of Toddlers
- Feasibility Study -

End of Data Collection Interview

Date: ____ / ____ / _______ Time: _____ : _____

Interviewer: ____________________ Participant's Code: _______________

1. *Introduction:*

Thank you very much for taking part in this study! We would just like to ask you some final questions about how easy or difficult it was to wear the activity monitors, fill out the activity diary and the questionnaire, and how useful were the reminders, in order to improve this process for future participants.
If you don't mind, I will record our talk to make it easier to remember all the useful details you can tell us. I assure you that your name or any personal details will not be recorded, and we will use only your participant code in the interview and data analysis. Is that alright with you?

(In case mother/caregiver does not give consent to record: *That is no problem, in that case I will just take some notes.)*

2. Questions about the activity monitor:

2.1. Was the activity monitor difficult for:

2.1.1. Your child to wear? ☐ YES ☐ NO

If YES - Could you tell me a bit more about it?

____

____

____

____

2.1.2. You to wear? ☐ YES ☐ NO

If YES - Could you tell me a bit more about it?

____

____

____

____

2.1.3. Your husband/partner to wear? ☐ YES ☐ NO

If YES - Could you tell me a bit more about it?

____

____

____

____

3. Questions about the Activity Diaries,

3.1. Was it difficult filling out the activity diary?

☐ YES ☐ NO

If YES - Could you tell me why?

☐ Understanding how to fill out the diary?

☐ Remembering to fill out the diary daily?

☐ Takes too much time to fill out?

☐ Other reason? ____

____

____

____

____

4. Questions about the reminders to wear the activity monitors:

4.1. What type of reminder did you have:

☐ Magnets ☐ Daily text message

4.2. Did you find it helpful in reminding you to wear the activity monitors every day?

☐ YES ☐ NO

5. Questions about the questionnaire:

5.1. Did you find the questionnaire difficult to answer?

☐ YES ☐ NO

If YES - Could you tell me why?

☐ Did not understand some questions? Which ones? ___________________________

____

☐ Did not understand some words? Which ones? ______________________________

____

☐ Takes too much time to answer?

☐ Other reason? ____

____

If NO – Did you find any words hard to understand?

☐ YES ☐ NO

If YES – Can you tell me which ones?____

____

6. Suggestions

6.1. Do you have any suggestions to make it easier to wear the accelerometers, fill out the activity diaries, understand the questionnaire, or anything you think we could have done differently?

____

____

____

____

____

7. Would you say this week of wearing the monitors was a typical week, or was it more active or less active than usual? (*If more/less* → Why?)

____

____

____

____

____

8. Lastly, did your child have any illness that caused diarrhoea, vomiting or loss of appetite during the past week?

☐ YES ☐ NO
